# Family functioning and mobile phone addiction in university students: Mediating effect of loneliness and moderating effect of capacity to be alone

**DOI:** 10.3389/fpsyg.2023.1076852

**Published:** 2023-02-09

**Authors:** Guan-Ru Li, Jian Sun, Jia-Nuo Ye, Xiao-Hui Hou, Ming-Qiang Xiang

**Affiliations:** ^1^Graduate School, Guangzhou Sport University, Guangzhou, China; ^2^School of Athletic Training, Guangzhou Sport University, Guangzhou, China; ^3^School of Sport and Health, Guangzhou Sport University, Guangzhou, China

**Keywords:** mobile phone addiction, family functioning, capacity to be alone, loneliness, moderated mediation model

## Abstract

**Background:**

With the increasing popularity of smartphones, mobile phone addiction in university students has attracted widespread societal attention. Previous studies showed that family functioning and mobile phone addiction are related. However, the potential mechanisms involved in this relationship are unknown. This study examined the mediating effect of loneliness and the moderating effect of capacity to be alone on the relationship between family functioning and mobile phone addiction.

**Methods:**

A total of 1,580 university students were recruited. A cross-sectional study design and online questionnaire survey were employed to measure demographic variables, family functioning, loneliness, capacity to be alone, and mobile phone addiction in university students.

**Results:**

Family functioning is a significantly negative predictor of mobile phone addiction in university students, and loneliness has a mediating effect on the relationship between family functioning and mobile phone addiction. The capacity to be alone has moderating effects on the relationship between family functioning and loneliness and between family functioning and mobile phone addiction, and these correlation is stronger in university students with a low capacity to be alone.

**Conclusion:**

The moderated mediation model in this study improves understanding of the correlation between family functioning and mobile phone addiction in university students. Education professionals and parents should pay particular attention to family functioning in mobile phone addiction, particularly university students with low capacity to be alone.

## 1. Introduction

With technological advancement, mobile phone usage has become more and more widespread. For example, the number of mobile phone users in China reached 987 million in December 2020, while the ratio of Internet users who surf the Internet using mobile phones was 99.7%, and these numbers are continuously increasing (China Internet Network Information Center, 2021). Smartphones are ubiquitous, and they can be used as mobile communication devices, Internet portals, social network platforms, personal organizers, and even mobile banks. Thus, smartphones have become part of the mainstream lifestyle in modern society (Lian et al., 2021). However, although mobile phones provide convenience, people are spending more and more time on mobile phones to satisfy certain needs, and some even cannot live without their mobile phones, resulting in mobile phone addiction (Kwon et al., 2013). Young people, particularly university students, are prone to mobile phone addiction. Compared with older social groups, university student populations are not emotionally mature and lack self-control (Long et al., 2016). In addition, most university students today are “data natives” and are born into a world where mobile phones are ubiquitous. These environmental factors cause them to be more prone to mobile phone addiction (Li L. et al., 2018; Li et al., 2020). Mobile phone addiction is a kind of behavioral addiction which has both similarities and differences with drug addiction. Using the concept of technology addiction as a reference, the researchers believe that mobile phone addiction is a non-biochemical (behavioral) impulse control disorder involving human-computer interaction (Leung, 2008). In addition, some studies have confirmed that mobile phone addiction is the users’ non-adaptive dependence and compulsive use of mobile phones (Zou et al., 2017). A meta-analysis showed that the incidence of mobile phone addiction in university students in China is around 23% (Tao et al., 2018). Therefore, there is a need to understand the risk factors and potential mechanisms of mobile phone addiction to better prevent and treat mobile phone addiction in university students.

The bioecological model states that the family is the most direct and influential factor in the ecological environment during the growth of an individual (Bronfenbrenner and Ceci, 1994). A poor family environment may obstruct the healthy physical and mental development of an individual. Among many family environmental factors, family functioning has an extremely important role. Family functioning refers to the overall quality of family life. A good family should have two important characteristics: cohesion and adaptability. Cohesion refers to emotional bonding that family members have toward one another, while adaptability refers to the ability of the family system to adapt to the environment and develop (Olson, 2000). If an individual perceives that there is a lack of emotional communication between family members and that family cohesion is low when he/she is growing up, then that individual will seek emotional support from the external world (such as the Internet; Chng et al., 2015). In addition, if family members lack the adaptability to solve problems together, then adolescents are prone to abnormal behavior and behavioral addiction when they are growing up (Li J. et al., 2018). Inspired by this theory, many studies have examined the role of family functioning in mobile phone addiction in adolescents. The results have shown that a lack of family functioning is an important predictor of mobile phone addiction in adolescents (Kim et al., 2018; Liu et al., 2020).

It should be stated that adolescents were the study subjects in most of the aforementioned studies, and very few studies examined the relationship between family functioning and mobile phone addiction in university students. In actuality, although the campus environment plays an important role in the lives of university students, family functioning still has important effects on the physical and mental health of university students (Yan et al., 2014; Zhai et al., 2016). More importantly, previous studies have paid less attention to the potential mediating effects and moderating mechanisms between family functioning and mobile phone addiction. This means that the mechanism through which family functioning affects mobile phone addiction is still unknown. Therefore, this study proposed a moderated mediation model to explain the intrinsic mechanisms between family functioning and mobile phone addiction. As loneliness is considered to be associated with psychological health problems and risky behavior in normal populations (Stickley and Koyanagi, 2016; Klein et al., 2021), this study uses loneliness as a mediating variable to analyze the relationship between family functioning and mobile phone addiction in university students. In addition, the capacity to be alone has been proven to be an effective buffer that can alleviate the effects of adverse external factors on risky behavior in individuals (Larson and Lee, 1996; Lian et al., 2021). Therefore, the capacity to be alone is used as a moderating variable in this study to examine the direct and indirect effects of family functioning on loneliness and mobile phone addiction.

### 1.1. Mediating effect of loneliness

Loneliness means that belongingness, a basic need of an individual, is not satisfied by the individual’s environment, resulting in a perception of social isolation or social disconnect (Hughes et al., 2004; Hawkley and Cacioppo, 2010). Loneliness is a subjective feeling that is not necessarily related to the number of times that one is alone but is related to the quality of interpersonal relationships (Hughes et al., 2004). Interpersonal relationship is the intimacy, satisfaction and trust that an individual can gain from others or a group after communicating their feelings, thoughts and emotions to them. The core experience of relationships is being socially isolated and lacking connections or being connected to a social group (Willems et al., 2020). As a result, a complex feeling of loneliness can arise when a person’s social needs are not fully met. In recent years, more and more individuals have experienced loneliness. According to different samples and measurement methods, around 11–40% of the general population experiences varying degrees of loneliness (Hawkley and Capitanio, 2015; Stickley and Koyanagi, 2016). Loneliness is a risk factor that causes poor physical and mental health and poor quality of life. This phenomenon has attracted widespread attention from researchers (Tzouvara et al., 2015; Klein et al., 2021). Many behavioral addiction studies show that loneliness is significantly positively correlated with excessive mobile phone usage and is an important predictor of mobile phone addiction (Tan et al., 2013; Jafari et al., 2019; Li et al., 2021). In addition, family functioning is one of the factors affecting loneliness in individuals. A study showed that family functioning is significantly negatively correlated with loneliness, and low family functioning is predictive of high loneliness (Sharabi et al., 2012).

With in-depth study, loneliness was found to play a mediating effect on the relationship between the environment and physical and mental health in individuals. For example, previous studies found that loneliness has a mediating effect on the relationship between child abuse and mobile phone addiction in adolescents (Ma et al., 2020), on the relationship between family cohesion and children’s effort (Feldman et al., 2018), and on the relationship between family functioning and psychological health in secondary vocational students (Pan et al., 2021). As family functioning has a strong direct effect on loneliness, and loneliness is a predictor of mobile phone addiction, this suggests that loneliness has a mediating effect. Therefore, this study hypothesizes that loneliness may have a mediating effect on the relationship between family functioning and mobile phone addiction.

### 1.2. Moderating effects of capacity to be alone

The capacity to be alone was first studied by psychologist Winnicott (1958). He found that this capacity not only has direct deterministic effects on improvement in a patient’s condition but is also a marker of mature emotional development in an individual. Although Larson and Lee (1996) classified solitude as involuntary solitude and constructed solitude, researchers usually use “capacity to be alone” to mean the ability of an individual in solitude to handle stress and perceive emotional comfort (Larson and Lee, 1996; Lian et al., 2021). Individuals with a high capacity to be alone may benefit the most from solitude, as they have better psychological adaptability and a positive and healthy lifestyle, including a negative correlation with depression and physical symptoms and a positive correlation with satisfaction with life (Larson and Lee, 1996; Cramer and Lake, 1998; Detrixhe, 2011). More importantly, a study showed that solitude has both cognitive functions and emotional functions, as it allows an individual to assess the adverse situation he/she is facing and provides opportunities to establish positive emotions (Winnicott, 1958; Lian et al., 2021). Hence, the capacity to be alone may have positive effects on psychological adaptation in an individual through cognitive and emotional processes.

In addition, a study by Wu and Chen (2006) found that the capacity to be alone can alleviate the adverse effects of objective life stress on psychological health. Compared with individuals with a high capacity to be alone, the effects of objective life stress are greater on individuals with a low capacity to be alone. Lian et al. (2021) showed that the capacity to be alone has moderating effects on the relationship between mobile phone addiction and psychological distress and the relationship between mobile phone addiction and rumination. Considering that a lack of family functioning will increase loneliness in individuals (Sharabi et al., 2012) and increase the risk of mobile phone addiction (Liu et al., 2020), while the capacity to be alone has positive effects (Winnicott, 1958) on psychological adaptation and healthy behavior through cognitive and emotional processes. Given that the ability to be alone can alleviate the adverse impact of life pressure on individual mental health, it can be inferred that people with strong ability to be alone have better mental ability to adapt to and bear the pressure brought by life when facing difficulties. Therefore, the capacity to be alone can alleviate both the effects of a lack of family functioning on loneliness and the effects of a lack of family functioning on mobile phone addiction. This means that the capacity to be alone has moderating effects on the relationship between family functioning and loneliness and the relationship between family functioning and mobile phone addiction.

### 1.3. The present study

**Figure 1:**
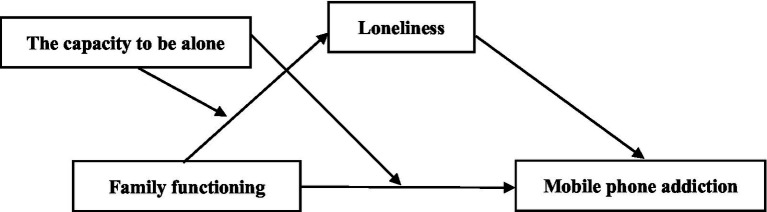
The proposed moderated mediation model.

The objective of this study is to examine the relationship between family functioning and mobile phone addiction in university students and further examine the mediating and moderating mechanisms involved to construct a moderated mediation model (as shown in Figure 1). Specifically, we proposed 3 study hypotheses: (1) Family functioning is a significant negative predictor of mobile phone addiction. (2) Loneliness has a mediating effect on the relationship between family functioning and mobile phone addiction. (3) The capacity to be alone has moderating effects on the relationship between family functioning and loneliness and the relationship between family functioning and mobile phone addiction.

## 2. Methods

### 2.1. Participants and processes

Convenience sampling was employed, and students from 5 universities in Guangzhou in southern China were recruited as study subjects. In order to ensure sample diversity, students were recruited from different types of universities, of which 3 were key universities (Cultivate talents in multiple fields) and 2 were normal universities (Specialized in training talents in the field of education). The study period was from January to March 2021. A questionnaire platform^1^ was used to design online questionnaires. Local lecturers distributed hyperlinks or QR codes of the questionnaire to university students on WeChat for them to fill in. After the student entered the main page of the survey, an online informed consent form was displayed. If the student did not disagree with the survey objective in the informed consent form, he/she clicked “Next” to start the survey. The student could stop the survey if he/she objected to it. This Internet-based online questionnaire survey was completely anonymous, voluntary, and non-commercial. For this study, we invited 2,215 university students to participate in the survey, of whom 1,633 completed the questionnaire. The response rate was 73.72%. After removing 53 questionnaires with short answering durations (<120 s) or those with significant abnormalities, 1,580 valid questionnaires were collected. The ratio of male to female subjects was 50% each, the age range was 18–27 years, and the mean age was 20.38 ± 1.98 years.

### 2.2. Measurements

#### 2.2.1. Demographic variables

As previous studies showed that the age, gender, family structure, and family financial situation of university students are correlated with mobile phone addiction (Zulkefly and Baharudin, 2009; Long et al., 2016), these demographic variables were controlled in the subsequent analysis. Specifically, the variables included gender (1 = male, 2 = female), age, whether the subject was an only child (1 = yes, 2 = no), family structure (1 = both parents present, 2 = reconstituted, 3 = single parent), living with whom (1 = parents, 2 = 1 of the parents, 3 = other family members or alone), and place of residence (1 = urban, 2 = rural). The subjects were required to report the socioeconomic status of their family, including the education levels of their parents and mean family monthly income. In the analysis, the family socioeconomic status was converted to a factor score (mean = 0, standard = 1). The higher the score, the higher the family socioeconomic status.

#### 2.2.2. Family functioning

The Family Adaptability and Cohesion Evaluation Scale (FACES II) was used to assess family functioning (Olson et al., 1983). This scale was translated into the Chinese version (FACESII-CV) and was verified to have good validity and reliability (Fei et al., 1991). The Chinese version (FACESII-CV) is suitable for Chinese subjects and has good validity and reliability (He et al., 2021). The scale contained 30 questions, including adaptability and cohesion dimensions. A 6-point Likert scale (1, almost never, 6: almost always) was used for all items. The higher the score, the better the family cohesion and the stronger the adaptability. The Cronbach’s *α* coefficient of this scale was 0.965.

#### 2.2.3. Loneliness

The UCLA loneliness scale developed by Russell et al. (1980) was used to measure loneliness. The Chinese translated version of this scale is widely used in China and has good validity and reliability (Li et al., 2021; Pan et al., 2021). The scale consists of 20 questions rated on a 4-point Likert scale (1: never, 4: often). The higher the score, the higher the level of loneliness. The Cronbach’s *α* coefficient of this scale was 0.947.

#### 2.2.4. Capacity to be alone

The Chinese version of the capacity to be alone scale (Wu and Chen, 2006) was used to measure capacity to be alone and was revised based on the original version (Larson, 1990). This scale has been used on Chinese students and has high validity and reliability (Lian et al., 2021). The scale consists of two mutually correlated 10-item scales, the solitude coping and solitude comfort scales. A 4-point Likert scale was used (1: never, 4: always). The Cronbach’s *α* coefficient of this scale was 0.951.

#### 2.2.5. Mobile phone addiction

The Chinese version of the smartphone addiction scale (SAS-SV) formulated by Kwon et al. (2013) and translated into Chinese by Xiang et al. (2019) was used to measure mobile phone addiction. Prior study showed that SAS-SV is also suitable for college students (Zhao et al., 2022). This scale has a single dimension and includes 10 questions. A 6-point Likert scale (1, extremely disagree, 6: extremely agree) was used. The higher the score, the greater the severity of mobile phone addiction. The Cronbach’s *α* coefficient of this scale was 0.895.

### 2.3. Data analysis

All data analysis was performed using SPSS 28.0. A value of *p* of 0.05 indicated statistical significance. First, frequency (percentage) or mean (standard deviation) were used to calculate demographic variables, including gender, age, whether the subject was an only child, family structure, living with whom, and family socioeconomic status. Independent *t*-test (two-tailed), One-way ANOVA or Pearson’s correlation analysis were used to examine the relationship between these demographic variables and mobile phone addiction based on the study objective and data type. Second, we entailed descriptive and Pearson’s correlation analyses to examine the results of the four surveys. Third, we used the SPSS PROCESS macro (model 8) proposed by Hayes (2013) to validate the moderated mediation model. A bootstrapping procedure was selected with 5,000 bootstrap samples used to calculate bias corrected 95% confidence intervals (CIs). A significant effect was considered to exist if the CIs did not include zero. In addition, all potential significant interactions were analyzed using simple gradients (Toothaker, 1994).

## 3. Results

### 3.1. Descriptive statistics and comparative analysis

Table 1 shows that a total of 1,580 subjects were included in the data analysis, and the ratio of male and female subjects was 50% each. In this population, 577 (36.5%) of university students were the only child, 782 (49.5%) had families that lived in urban areas, and 798 (50.5%) had families that lived in rural areas. With regard to family structure, 1,128 (71.4%) lived in intact families, 228 (14.4%) lived in divorced families, and 224 (14.2%) lived in reconstituted families. Furthermore, 776 (48.5%) university students lived with both parents, 468 (29.6%) university students lived with 1 parent, and 346 (21.9%) university students lived with other relatives or alone. Comparative analysis found that family socioeconomic status was negatively correlated with mobile phone addiction in university students (*r* = −0.156, *p* < 0.001). Mobile phone addiction was higher in only children than in subjects with siblings (*t* = 5.775, *p* < 0.001) and higher in university students from urban families than in those from rural families (*t* = 3.044, *p* = 0.002). Meanwhile, mobile phone addiction was lower in university students living with both parents compared with those who lived with one parent or other relatives (or alone) (*p* < 0.05).

**Table 1:** Participants’ sociodemographic characteristics and smartphone addiction scores.

	Mean ± SD or n (%)	SAS-SV scores Mean ± SD	*r/t/F*	*p*
**Age**	20.38 ± 1.98		*r* = 0.009	0.71
**Socioeconomic status**	0 ± 1		*r* = −0.156	<0.001
**Gender**			*t* = 1.783	0.082
Female	790 (50%)	40.66 ± 10.29		
Male	790 (50%)	39.71 ± 11.39		
**Only child**			*t* = 5.775	<0.001
Yes	577 (36.5%)	42.24 ± 11.774		
No	1,003 (63.5%)	39.00 ± 10.118		
**Family residence**			*t* = 3.044	0.002
City	782 (49.5%)	41.02 ± 11.01		
Rural	798 (50.5%)	39.36 ± 10.66		
**Family structure**			*F* = 0.048	0.958
Integrated	1,128 (71.4%)	40.21 ± 10.87		
Divorced	228 (14.4%)	40.28 ± 9.79		
Reconstituted	224 (14.2%)	39.99 ± 11.83		
**Living with parent(s)**				*F* = 3.718	0.025
Both parents (1)	766 (48.5%)	39.43 ± 10.98	1 < 2,1 < 3	
One parent (2)	468 (29.6%)	40.75 ± 10.88		
Other relatives or alone (3)	346 (21.9%)	41.09 ± 10.48		

### 3.2. Common method bias test

Harman’s single factor method was used for common method bias test (Harman, 1960). The test result showed that the maximum factor variance interpretation rate is 21.2%, which is below the threshold value of 40%. It was thus inferred that there were no serious common method bias problems in this study.

### 3.3. Correlation analyses

Table 2 depicts correlations between the metric variables family functioning, the capacity to be alone, loneliness and mobile phone addiction. Family functioning was negatively associated with loneliness and mobile phone addiction, but positively associated with the capacity to be alone; The capacity to be alone and loneliness was negatively related; Loneliness and mobile phone addiction was were positively related.

**Table 2:** Descriptive statistics of means, SD, and Pearson’s correlations.

	M	SD	1	2	3	4
1. Family functioning	99.84	16.17	1			
2. The capacity to be alone	51.75	5.044	0.092**	1		
3. Loneliness	49.56	4.985	−0.087**	−0.113**	1	
4. Mobile phone addiction	40.18	10.862	−0.220**	−0.031	0.190**	1

**p < 0.01.

### 3.4. Moderating and mediating effect test

**Figure 2:**
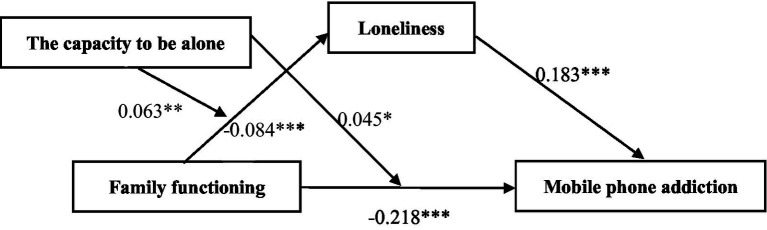
The path coefficients in the moderated mediation model. ***p < 0.001, **p < 0.01, *p < 0.05.

The SPSS PROCESS macro (model 8) proposed by Hayes (2013) was used to test the moderated mediation model. The results are shown in Table 3 and Figure 2. The mediation variable model [*F*(12,1,567) = 16.08, *R*^2^ = 0.118, *p* < 0.001] and dependent variable model [*F*(13,1,566) = 34.83, *R*^2^ = 0.21.2, *p* < 0.001] reached statistical significance. After controlling for demographic variables, family functioning was found to be a significant negative predictor of mobile phone addiction (*β* = −0.218, *p* < 0.001), supporting hypothesis 1. At the same time, family functioning was a significant negative predictor of loneliness (*β* = −0.084, *p* < 0.001), while loneliness was a significant positive predictor of mobile phone addiction (*β* = 0.183, *p* < 0.001). More important, the moderated mediation index (a direct quantification of the linear association between the indirect effect and the putative moderator of that effect) was significant [index = −0.041, Standard error (SE) = 0.009, 95% CI = −0.072 to −0.010], showing that family functioning indirectly affects mobile phone addiction through loneliness. This result provides strong evidence for loneliness being a significant mediator of the relationship between family functioning and mobile phone addiction and supports hypothesis 2.

**Table 3:** Testing for the moderated mediation effect.

Model	Outcome	Predictiors	*R*	*R^2^*	*F*	*β*	*t*	95%CI
Mediator variable	Loneliness		0.34	0.118	16.08***			
		Family functioning				−0.084	−3.393***	[−0.133, −0.036]
		The capacity to be alone				−0.080	−3.236***	[−0.128, −0.031]
		Family functioning × The capacity to be alone				0.063	2.775**	[0.019, 0.108]
Dependent variable	Mobile phone addiction		0.46	0.212	34.83***			
		Family functioning				−0.218	−8.958***	[−0.266, −0.171]
		Loneliness				0.183	7.422***	[0.135, 0.232]
		The capacity to be alone				−0.020	−0.813	[−0.067, 0.028]
		Family functioning × The capacity to be alone				0.045	1.987*	[0.001, 0.088]

*N* = 1,580. Unstandardized regression coefficients are reported. Bootstrap sample size = 5,000. CI, confidence interval. ***p < 0.001, **p < 0.01, *p < 0.05.

In order to further validate hypotheses 3, we further analyzed the interactions between two variables in this study. The results showed that the interaction between family functioning and capacity to be alone in the mediating variable model is a significant positive predictor of loneliness (*β* = −0.063, *p* < 0.01). In the dependent variable model, the interaction between family functioning and capacity to be alone is also a significant positive predictor of mobile phone addiction (*β* = 0.045, *p* < 0.05). The interactions in the two models were significant, showing that the capacity to be alone has moderating effects on the relationship between family functioning and loneliness and the relationship between family functioning and mobile phone addiction. This supports hypothesis 3.

**Figure 3:**
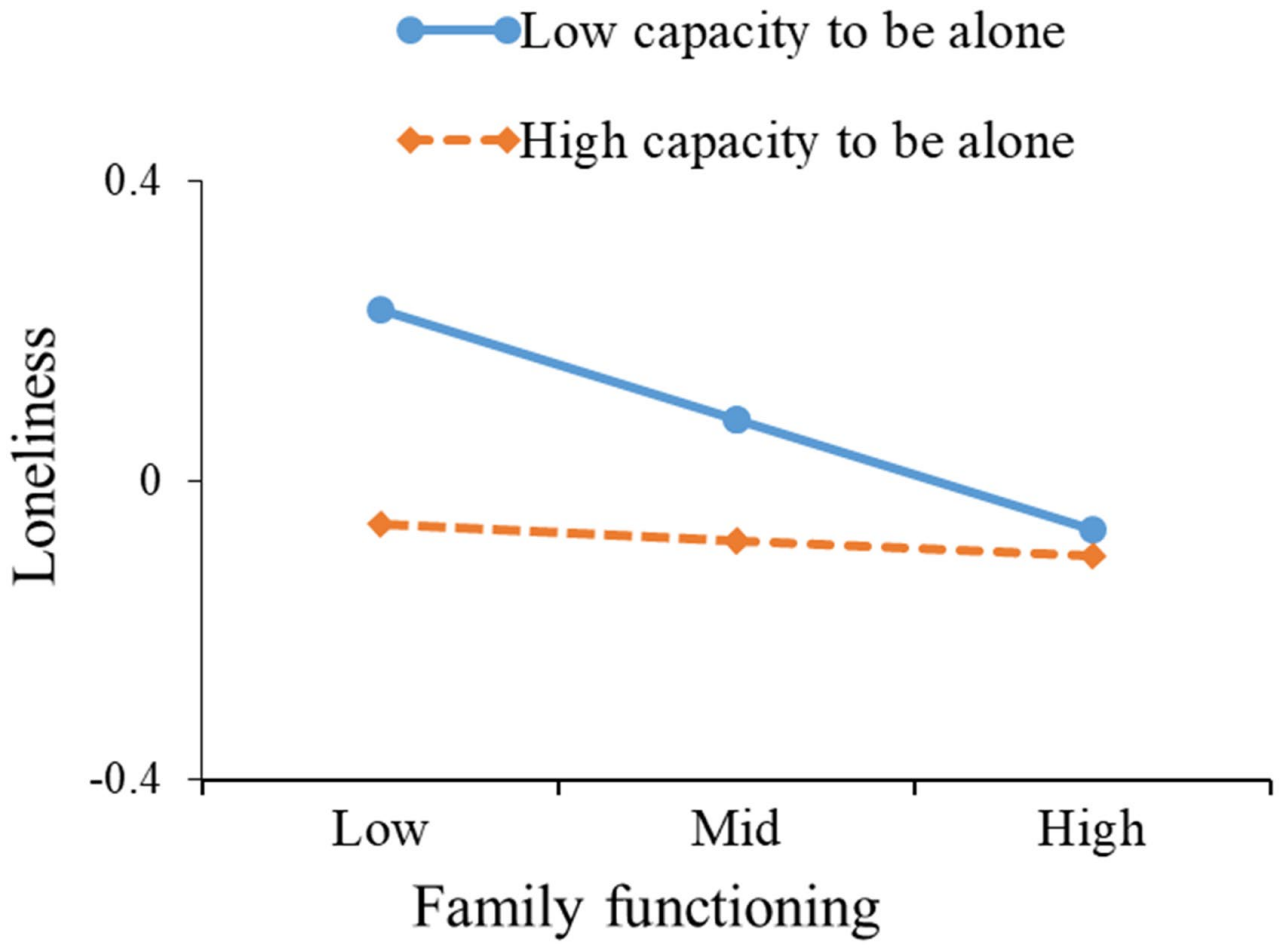
The capacity to be alone moderates the relation between family functioning and loneliness.

**Figure 4:**
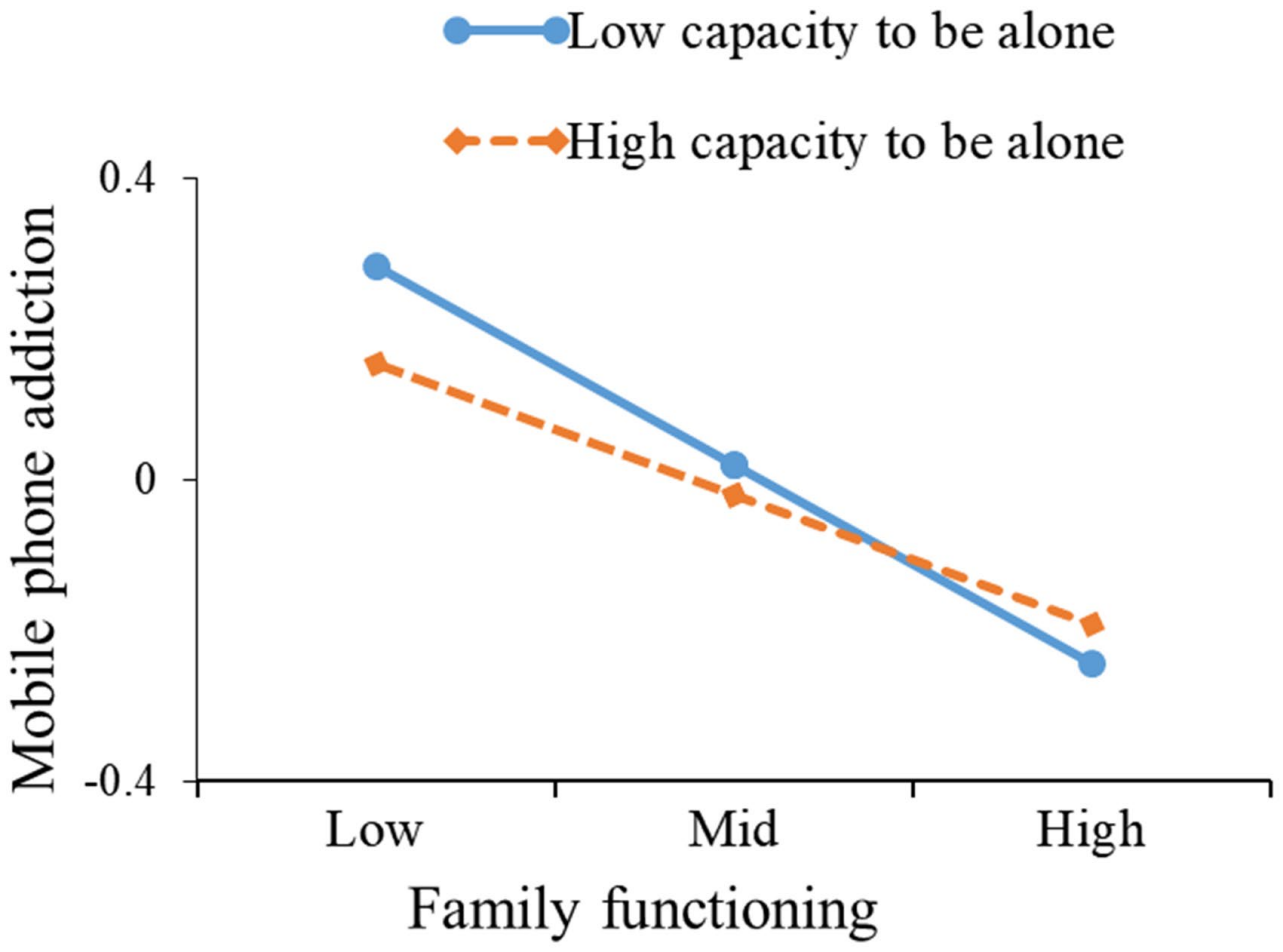
The capacity to be alone moderates the relation between family functioning and mobile phone addiction.

Furthermore, this study used simple slope analysis to further clarify these significant interactions and examine whether the slope of the low capacity to be alone group (1 standard deviation lower than the mean) was higher than that of the high capacity to be alone group (one standard deviation higher than the mean). The results are shown in Table 4 and Figures 3, 4. From Table 4 and Figure 3, it can be seen that in university students with a low capacity to be alone, family functioning affected loneliness (*β* = −0.027, 95% CI = −0.051 to −0.008, excluding 0), but family functioning did not affect loneliness in university students with a high capacity to be alone (*β* = −0.004, 95% CI = −0.027 to 0.018, including 0). From Table 4 and Figure 4, it can be seen that the effect of family functioning on mobile phone addiction was higher in students with a low capacity to be alone (*β* = −0.263, 95% CI = −0.327 to −0.199, excluding 0) than in students with a high capacity to be alone (*β* = −0.174, 95% CI = −0.239 to −0.108, excluding 0). The presence of interactions between family functioning and capacity to be alone means that university students with high capacity to be alone experience less loneliness or mobile phone addiction regardless of their family functioning status, while university students with low capacity to be alone will experience more loneliness or greater mobile phone addiction.

**Table 4:** Conditional effect analysis at values of the self-control variable.

Conditional direct effect analysis at values of the capacity to be alone (M ± SD)				
	β	SE	LLCI	ULCI
M-SD	−0.263	0.033	−0.327	−0.199
M	−0.218	0.024	−0.266	−0.171
M + SD	−0.174	0.033	−0.239	−0.108
Conditional indirect effect analysis at values of the capacity to be alone (M ± SD)				
	β	BootSE	BootLLCI	BootULCI
M-SD	−0.027	0.011	−0.051	−0.008
M	−0.015	0.006	−0.029	−0.006
M + SD	−0.004	0.011	−0.027	0.018

*N* = 1,580. Bootstrap sample size = 5,000. LL, low limit; CI, confidence interval; UL, upper limit.

## 4. Discussion

The aim of this study was to examine the effects of family functioning on mobile phone addiction in university students and the effects of loneliness and capacity to be alone on the relationship between family functioning and mobile phone addiction. Our study found that family functioning is a significantly negative predictor of mobile phone addiction in university students, and loneliness has a mediating effect on the relationship between family functioning and mobile phone addiction. More importantly, the capacity to be alone has moderating effects on the relationship between family functioning and loneliness and the relationship between family functioning and mobile phone addiction.

### 4.1. Effects of family functioning on mobile phone addiction in university students

The results of the study showed that family functioning is a negative predictor of mobile phone addiction in university students. This means that good family functioning, such as harmonious and loving family relationships, is associated with low mobile phone addiction. However, university student growing up in dysfunctional family, defined as “family in which relationships or communication are impaired and members are unable to achieve intimacy and self-expression” (Munro, 1985), such as outbreak of conflicts within the family, breakdown of emotional connection, lack of communication between family members and abnormal behavior of some family members, are prone to mobile phone addiction. Due to the lack of emotion, college students might not find a vent for their emotions, and the family atmosphere is dignified, so they tend to place their emotions on materials, such as mobile phones. With the convenience of mobile devices and the ability to meet most of people’s life needs, college students might seek spiritual comfort and temporarily forget their family’s misfortune. Specifically, parents’ low emotional involvement may lead college students to overuse cell phones as a strategy for coping with the resulting psychological distress. This matches the results of previous studies on adolescents (Kim et al., 2018; Liu et al., 2020). This result supports the McMaster family functioning model (Miller et al., 2000) and family functioning process model (Skinner et al., 2000). These two models state that the realization of various family functions will directly affect the physical and mental health and emotional behavior of individuals. If family functioning is successfully achieved and a good state is reached, then the health behavior of family members is improved. Otherwise, family members are prone to risky behavior, such as mobile phone addiction and Internet addiction (Chng et al., 2015; Liu et al., 2020).

The family functioning model holds that the basic function of the family is to provide good environmental conditions for developing physiological, psychological, and social health in family members (Pan et al., 2021). This means that family factors and individual health behavior problems are intimately associated. Although the life of university students includes the family and campus environment, they are still emotionally immature and are still deeply affected by family functioning. Good family functioning will help alleviate anxiety and unease and enable students to better cope with learning and social life events, thereby improving their psychological and behavioral problems. Conversely, a child growing up in a family environment that lacks cohesion and adaptability is unable to experience enough caring. Therefore, they may seek that missing emotion through social networks as a form of compensation (Chng et al., 2015). Smartphones are convenient tools for online socializing and obtaining emotional comfort. Therefore, the risky behavior of seeking emotional support on mobile phones may extend to campus life.

### 4.2. Moderating and mediating effects

Our study shows that loneliness has a mediating effect on the relationship between family functioning and mobile phone addiction in university students. This means that family functioning indirectly affects mobile phone addiction in university students through loneliness. This provides a new perspective on how family functioning can affect mobile phone addiction. Many studies have found that family functioning is associated with loneliness (Sharabi et al., 2012; Pan et al., 2021). This shows that family functioning is an important factor that affects loneliness in university students. Poor family functioning will cause family members to have poor interpersonal skills, poor communication, emotional problems, and interpersonal communication barriers. This ultimately results in tense interpersonal relationships and increases loneliness (Yang et al., 2011). More importantly, the incidence of mobile phone addiction is higher when loneliness is greater (Tan et al., 2013; Jafari et al., 2019; Li et al., 2021). However, in a good family functioning environment, university students can positively communicate with family members to obtain emotional support and decrease loneliness, thereby decreasing the occurrence of mobile phone addiction. In addition, the results of this study also support the basic psychological need theory (Vansteenkiste et al., 2020). This theory states that satisfying the basic psychological needs of autonomy, competence, and belongingness are basic motivations for behavior and basic assurances for growth in individuals. If the basic psychological needs of an individual cannot be satisfied, this will produce a strong desire to satisfy these needs, thereby forcing the individual to turn to other scenarios that can satisfy these needs (Deci and Ryan, 2000; Vansteenkiste et al., 2020). Specifically, poor family functioning environments will result in poor cognition in university students, thereby resulting in more negative emotions and decreased social support. In order to satisfy their needs of love and belongingness, they will use mobile phones to enter a virtual world to cope with loneliness and receive emotional support.

An important finding of this study is the relationships of family functioning with the mediating variable loneliness and the dependent variable mobile phone addiction are both moderated by capacity to be alone. These effects are greater in individuals with a low capacity to be alone compared with those with a high capacity to be alone. This indicates that the capacity to be alone is a positive psychological, emotional, and behavioral factor that can effectively alleviate the adverse effects of low family functioning on loneliness and mobile phone addiction.

Previous studies showed that we are able to shift our attention from the external environment to the mind. During solitude, we are better able to understand and assess ourselves (Larson, 1990). University students with a high capacity to be alone may actively seek solitude to regulate and cope with loneliness caused by low family functioning. At the same time, they can distance themselves from emotional attachment to the virtual world through cognitive and emotional adjustment to cope with mobile phone addiction induced by low family functioning. Unfortunately, university students with a low capacity to be alone cannot benefit from solitude. When they are alone, they often spend more time on activities that distract their attention, and solitary feelings are described as anxious, lonely, and depressed (Long et al., 2003). Therefore, university students with a low capacity to be alone may be prone to loneliness and mobile phone addiction when family functioning is dysregulated, resulting in a vicious cycle.

### 4.3. Limitations and applications

Even though this study provided valuable findings on how family functioning affects loneliness and mobile phone addiction, the following limitations are present in this study. First, a cross-sectional design was used for this study. Even though the cross-sectional design is widely used in mobile phone addiction studies (Long et al., 2016; Liu et al., 2020; Lian et al., 2021), cross-sectional data cannot determine causality and individual developmental differences. Therefore, a longitudinal design can be used in future studies to better validate the moderated mediation model and examine the causal relationships between family functioning, loneliness, and mobile phone addiction. Second, the data in this study was self-reported by university students. Even though these self-reported scales have been verified to have good validity and reliability, other evaluation methods (such as parent evaluation) and objective records from mobile apps of time spent by university students on mobile phones can be used in future studies to validate this study model. Third, this study emphasizes the effects of family functioning on mobile phone addiction. As family functioning may be influenced by different cultures, it remains to be verified whether the results of this study could be generalized to other countries and cultures. In addition, the mobile phone functions that university students were addicted to were not examined. In the future, the effects of family functioning on specific mobile phone addiction behaviors can be further examined.

Despite its limitations, this study has important theoretical and practical significance. First, the study examined how a family factor (family functioning) and an individual factor (capacity to be alone) in university students interact with mobile phone addiction and expands on previous studies. The study results provide evidence that an interaction between environmental factors and individual factors jointly determines the psychological behavior adaptation in university students. Second, this study revealed moderating and mediating mechanisms between family functioning and mobile phone addiction. These findings can aid in better understanding of how and when family functioning is related to mobile phone addiction in university students. Third, this study provides important recommendations for prevention of and intervention in mobile phone addiction in university students. For example, parents can construct a supportive environment with open exchange, low family conflict, and positive interactions between family members for university students so that students can feel family intimacy and belongingness and decrease loneliness, thereby decreasing mobile phone addiction. Considering that the capacity to be alone is a protective factor of poor family functioning, loneliness, and mobile phone addiction, some psychological interventions, such as mindfulness training (Brown et al., 2007), could be employed to improve university students’ capacity to be alone, thereby decreasing the negative effects induced by a lack of family functioning.

## 5. Conclusion

Our study found that family functioning is a significant negative predictor of mobile phone addiction in university students, and loneliness has a mediating effect on the relationship between family functioning and mobile phone addiction. In addition, the capacity to be alone has moderating effects on the relationship between family functioning and loneliness and the relationship between family functioning and mobile phone addiction. This relationship is stronger in university students with a low capacity to be alone compared with university students with a high capacity to be alone. The moderation and mediation model in this study helps to better understand the correlation between family functioning and mobile phone addiction in university students. The effects of the interaction between an environmental factor (family functioning) and an individual factor (capacity to be alone) on mobile phone addiction in university students are emphasized.

## Data availability statement

The original contributions presented in the study are included in the article/supplementary material, further inquiries can be directed to the corresponding authors.

## Ethics statement

The studies involving human participants were reviewed and approved by the Ethics Committee of the Guangzhou Sport University. The patients/participants provided their written informed consent to participate in this study.

## Author contributions

X-HH, M-QX, and JS: conceptualization. G-RL and J-NY: data curation. G-RL, J-NY, and M-QX: methodology and writing – review and editing. X-HH and JS: supervision. G-RL and M-QX: writing – original draft. All authors contributed to the article and approved the submitted version.

## Funding

This research was funded by the National Social Science Foundation of China (grant number 18BTY055).

## Conflict of interest

The authors declare that the research was conducted in the absence of any commercial or financial relationships that could be construed as a potential conflict of interest.

## Publisher’s note

All claims expressed in this article are solely those of the authors and do not necessarily represent those of their affiliated organizations, or those of the publisher, the editors and the reviewers. Any product that may be evaluated in this article, or claim that may be made by its manufacturer, is not guaranteed or endorsed by the publisher.

## Footnotes

^1^ www.wjx.cn
